# The Baltimore College of Dental Surgery

**Published:** 1872-11

**Authors:** 


### EDITORIAL DEPARTMENT.

*The Baltimore College of Dental Surgery.*—The *thirty-third* annual session of this institution began on the 15th of October, with the regular opening of the Infirmary, and the delivery of the first of the preliminary lectures by Prof. P. H. Austen. The regular course of lectures commences on the 1st of November. This is the first session of the College in the new College Building, North Green Street, which has been fitted up in a manner superior to that of the old building, so many years occupied by this institution, and which is now known as the College of Physicians and Surgeons, a new medical college lately instituted in this city, being the third in Baltimore.

The present session of the Baltimore College of Dental Surgery, promises thus far by the large number of students present so early in the course, to be one of the most successful ones ever held. Never since the late war, prior to which this college ranked the highest in the number of dental students, have so many matriculated so early in the session, and the indications for a large class in 1872-73 are very encouraging.

By the death of the late Prof. Thomas E Bond, M. D., the College lost its oldest Professor, but owing to the creation of the new chair of Clinical Dentistry, the number of the faculty has not been reduced. Prof. Bond's chair has been divided as follows: to Prof. Noel, in connection with physiology, has been assigned dental pathology; to Prof. DeRosset, in connection with chemistry, materia medica; and to Prof. Gorgas, in connection with dental surgery, dental therapeutics.

The college now possesses a much larger Infirmary, with windows on every side, and also much easier of access, than the one in the old building, and in which a number of new, finely finished chairs have been placed. No pains will be spared to render the practical department one of the most prominent in the course of study; and the new location promises to more than double the number of Infirmary patients.

The Infirmary was opened in the new building in April last, and since that time it has been in successful operation.

